# Circular RNA circ_PVT1 induces epithelial-mesenchymal transition to promote metastasis of cervical cancer

**DOI:** 10.18632/aging.103679

**Published:** 2020-10-27

**Authors:** Hongping Wang, Meiling Wei, Yihua Kang, Jianqin Xing, Yinghui Zhao

**Affiliations:** 1 Department of Gynecology, Jinan City People’s Hospital, Jinan, China; 2 Department of Gynecology and Obstetrics, Yiyuan Chinese Medicine Hospital, Yiyuan, China; 3 Department of Gynecology and Obstetrics, Weihai Central Hospital, Weihai, China

**Keywords:** cervical cancer, gynecological malignant tumors, circRNA, miR-1286, EMT

## Abstract

Cervical cancer is one of the most common gynecological malignant tumors. At present, it has been confirmed that the occurrence and development of cervical cancer is related to human papillomavirus infection. As a new regulatory molecule and research hotspot, circRNA is abnormally expressed in tumors and other diseases, and is expected to become a new biomarker for diagnosis and prediction of tumor occurrence and development. In this research, bioinformatics analysis and RT-PCR analysis showed that hsa_circ_0009143 (circRNA_PVT1) was up-regulated in cervical cancer. Knockdown of circRNA_PVT1 inhibits the migration and invasion of cervical cancer cells and would prevent pulmonary metastasis. Overexpression of circRNA_PVT1 induced migration and invasion of cervical cancer cells, which would result in the promotion of pulmonary metastasis. Finally, we found that circRNA_PVT1 can induce EMT of cervical cancer cells via targeting miR-1286 by exosome pathway, which can be a novel mechanism of cervical cancer progression.

## INTRODUCTION

Cervical cancer (CC) is a tumor occurring in cervical epithelium, which is one of the most common malignant tumors in women in the world [1, 2]. It ranks second in malignant degree and incidence, and ranks fifth in fatality rate. The annual death toll and morbidity are 275,100 and 529,800 respectively [3]. The infection of high-risk human papillomavirus (HPV) is the main cause of cervical cancer [4, 5]. However, the occurrence of cervical carcinogenesis is a process of multi-gene and multi-stage abnormal regulation. In addition to HPV infection, there are a variety of genes that can be co-assisted or antagonized.

The DNA that can encode proteins in the human genome is only 20%, and the rest are called non-coding RNA (ncRNAs) [6]. They are not translated into proteins and are considered to be the dark matter of the genome, but it does not mean that they have no genetic information or biological functions. Previous reports have shown that ncRNAs is participated in serious of biological processes, cell proliferation, differentiation, apoptosis, metabolism and senescence, especially in post-transcriptional regulation [7, 8]. CircRNA is rare and was once considered a by-product of erroneous splicing [9, 10]. Later, it was reported that circRNA exists widely in animal and plant cells and tissues, and has many special biological characteristics, which attracted the attention of scientists at home and abroad [11]. At present, there are still many unknown biological functions of circRNA, so the research should be strengthened to make it widely used in various fields.

It has been found that the occurrence and development of cervical cancer is closely related to the abnormal expression of circRNA. The expression of total circRNA in hepatocellular carcinoma was analyzed by circRNA microarray, and it was stated that the expression of hsa_circ_0004018 decreased significantly, and the low level of hsa_circ_0004018 was correlated with the level of AFP and different stages of tumorigenesis and development. hsa_circ_0004018 can also affect the biological process of liver cancer through its interaction with miRNA [12]. It has been found that circRBM33 are closely related to the pathological stage and poor prognosis of patients with gastric cancer [13]. The interaction between circLARP4 and miR-424 affects the biological behavior of gastric cancer cells. Studies have shown that hsa_circ_0013958 may be a potential molecular marker for early diagnosis and prevention of lung adenocarcinoma [14]. It was reported that circRNA_PVT1 involved in progression of ovarian cancer [15].

Exosomes are small vesicles containing nucleic acids, proteins and lipids, which play a key role in intercellular communication by transmitting active biomolecules [16, 17]. Exosomes produced by tumor cells can produce pre-metastatic microenvironment in predetermined metastatic organs by inducing immunosuppression, fibrosis or inflammation [18]. Many ncRNAs were involved in regulating tumors via exosomes [19].

Under the action of external factors, the expression of the original epithelial marker molecules decreased, the intercellular connection decreased, while the interstitial marker molecules re-expressed or increased, and the morphology changed from cubic and polar distribution to cord type and random distribution, this phenomenon is called tumor cell Epithelial mesenchymal transformation (EMT) [20, 21]. The recognized markers are epithelial markers (E-cadherin) and increased level of (vimentin), which leads to the migration and invasion ability of cells. It was reported that circ-HIPK3/miR-338-3p promote cell development in cervical cancer, through regulating HIF-1α mediated EMT. Meng J et.al twist1 regulates vimentin through Cul2 to induce EMT in hepatocellular carcinoma [22]. Circ_0008305 could inhibit TGF-β-induced EMT and metastasis via regulating TIF1γ in NSCLC [23]. CircRNA MYLK accelerates bladder cancer development via regulating VEGFA [24]. In this article, we will explain the role of circRNA_PVT1 in cervical cancer.

## RESULTS

### Circ_PVT1 is upregulated in cervical cancer

**Figure 1:**
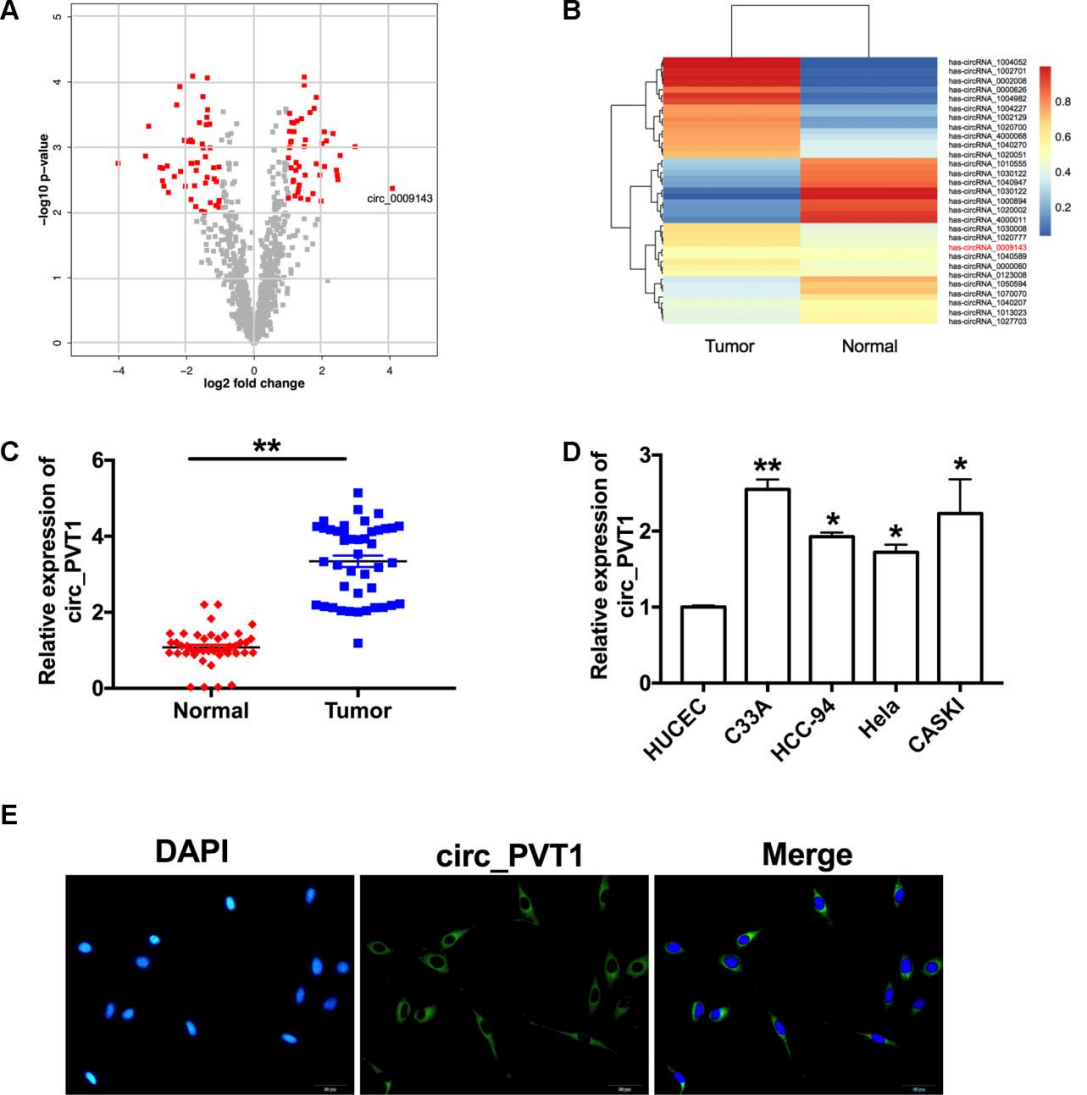
**Circ_PVT1 is upregulated in cervical cancer.** (**A**) The volcano plot of gene. (**B**) Heat map of microarray data was used to show the significant expression of circRNAs in tumor and normal tissue. (**C**) The expression of circ_PVT1 was detected in CC patients tumor tissue and normal tissues. n=43, **P<0.01. (**D**) RT-PCR assay was performed to measure the expression of circ_PVT1 in different CC cell lines (C33A, HCC-94, Hela and CASKI), HUCEC cell line was described as a control. n=10, *P<0.05, **P<0.01. (**E**) Representative photo of RNA fluorescence in situ hybridization for endogenous circ_PVT1 in C33A cells.

Volcano map and Chip data showed that circ_PVT1 (hsa_circ_0009143) was up-regulated in CC patients (Figure 1A and 1B). We detected the expression of circ_PVT1 in tumor and adjacent tissues from 43 CC patients. RT-PCR assays performed that, compare with adjacent group, circ_PVT1 was up-regulated in tumor tissues (Figure 1C). Then level of circ_PVT1 were also detected in different CC cell lines (C33A, HCC-94, Hela and CASKI), HUCEC cell line was described as a control. We observed that Circ_PVT1 showed different degrees of increase in CC cell lines, especially in C33A and CASKI cell lines (Figure 1D). RNA fluorescence in situ hybridization showed that endogenous circ_PVT1 was located in cytoplasm (Figure 1E).

### Knockdown of circ_PVT1 prevents migration and metastasis of cervical cancer cells

**Figure 2:**
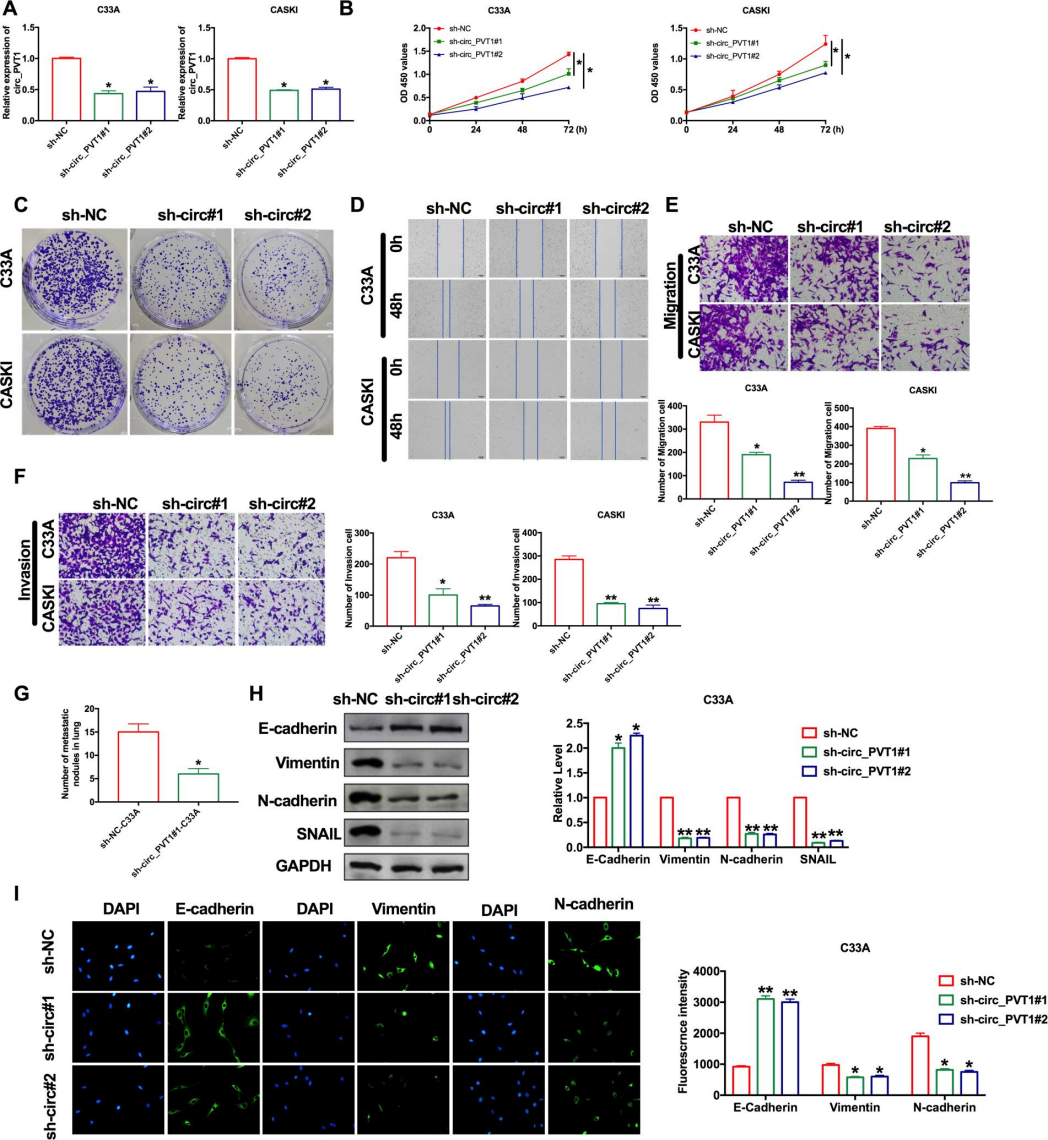
**Downregulation of circ_PVT1 inhibits migration and invasion of CC cells by regulating EMT.** (**A**) The transfection efficiency was verified by RT-PCR. n=8, *P<0.05. (**B**) The CCK8 assay was performed in cervical cancer cell lines. n=8, *P < 0.05. (**C**) Clone formation assays in cervical cancer cell lines. (**D**) Wound healing assay was carried out for migration. (**E**, **F**) The migration and invasion were explored by transwell. n=6, *P<0.05, **P<0.01. (**G**) The number of nodules was statistics for detecting lung metastasis. n=6, *P<0.05. (**H**) The expression of metastasis-associated protein, E-cadherin, Vimentin, N-cadherin and SNAIL were detected. n=6, *P<0.05, **P<0.01. (**I**) Immunofluorescence representative images of E-cadherin, Vimentin, N-cadherin. n=6, *P<0.05, **P<0.01.

The abnormal increase of circ_PVT1 indicated that it may be involved in the development of CC. Then we explored the function in CC by loss function of circ_PVT1. Transfection effective was verified by RT-PCR assay (Figure 2A). CCK-8 showed that the cell viability of CC cell lines decreased significantly after loss function of circ_PVT1 (Figure 2B). Clone formation assay highlighted the evidently reduced proliferation of CC cell lines treated with si-circ_PVT1 (Figure 2C). Wound healing assays showed that knockdown of circ_PVT1 prevented the migration of C33A and CASKI cells (Figure 2D). Transwell assays also showed the similar results (Figure 2E). We observed that sh-circ_PVT1 inhibited the invasion of CC cells by performing matrigel invasion assay (Figure 2F). Then we created stable low expression circ_PVT1C33A cell line (sh_PVT1_C33A) and negative control C33A cell line (sh_NC_C33A), mice were injection the cell via tail vain for lung metastasis assays. By calculating the number of pulmonary nodules, knockdown of circ_PVT1 significantly inhibited the lung metastasis (Figure 2G). EMT is a key step in tumor invasion and metastasis. Then we measured the EMT biomarker E-cadherin, Vimentin, N-cadherin and SNAIL in C33A cells. Western blot experiments revealed that sh-circ_PVT1 induced the up-regulated of E-cadherin and down-regulated of Vimentin, N-cadherin SNAIL (Figure 2H). Immunofluorescence assay once again confirmed the up-regulated of E-cadherin and down-regulated of Vimentin, N-cadherin by knockdown circ_PVT1(Figure 2I). In summary, knockdown of circ_PVT1 could inhibit the migration and metastasis of CC by blocking EMT program.

### Overexpression of circ_PVT1 induces migration and metastasis of cervical cancer cells

**Figure 3:**
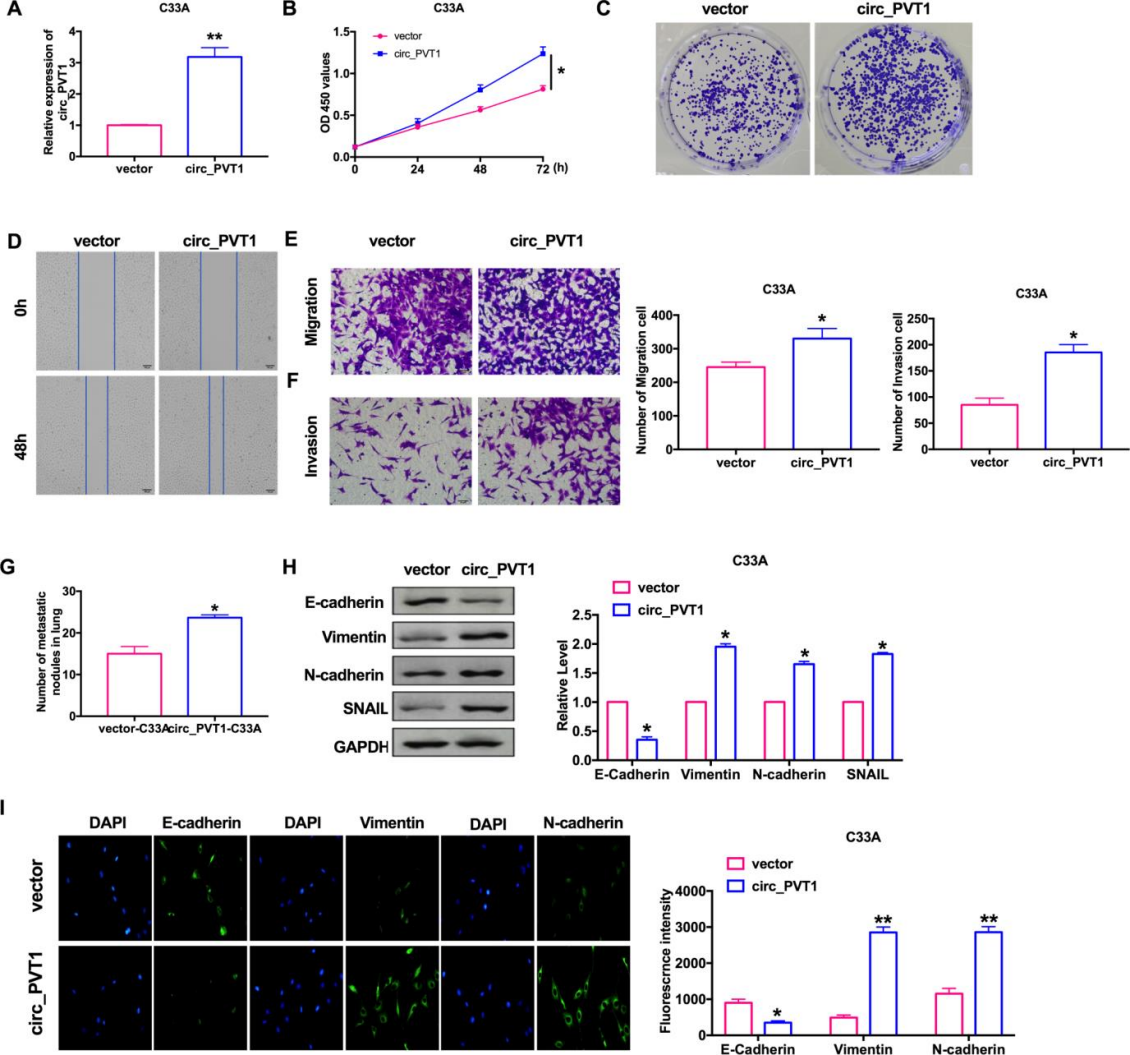
**Upregulation of circ_PVT1 induces migration and invasion of CC cells by regulating EMT.** (**A**) The transfection efficiency was verified by RT-PCR. n=6, **P<0.01. (**B**) The CCK8 assay was performed in cervical cancer cell lines. n=8, *P < 0.05. (**C**) Clone formation assays in cervical cancer cell lines. (**D**) Wound healing assay was carried out for migration. (**E**, **F**) The migration and invasion were explored by transwell. n=6, *P<0.05. (**G**) The number of nodules was statistics for detecting lung metastasis. n=6, *P<0.05. (**H**) The expression of metastasis-associated protein, E-cadherin, Vimentin, N-cadherin and SNAIL were detected. n=6, *P<0.05. (**I**) Immunofluorescence representative images of E-cadherin, Vimentin, N-cadherin. n=6, *P<0.05, **P<0.01.

For further explore the function of circ_PVT1, we constructed the plasmid for overexpression of circ_PVT1 (circ_PVT1), and vector plasmid was indicated as control (vector), the efficiency of plasmid was ensured by RT-PCR (Figure 3A). CCK-8 showed that the cell viability of C33A induced by forced function of circ_PVT1 (Figure 3B). Clone formation assay performed increased proliferation of C33A after circ_PVT1 transfection (Figure 3C). Wound healing and transwell assays performed that gain function of circ_PVT1 promoted migration of CC cells (Figure 3D and 3E). The invasion assay verified that overexpression of circ_PVT1 accelerated the metastasis of CC cells (Figure 3F). Accompanied by increasing number of nodules, circ_PVT1 aggrandized the lung metastasis (Figure 3G). Meanwhile, the down-regulated of E-cadherin and up-regulated of Vimentin, N-cadherin SNAIL after circ_PVT1 transfection revealed that circ_PVT1 could induce EMT course (Figure 3H and 3I).

### Circ_PVT1 binds directly to miR-1286 in C33A cells

**Figure 4:**
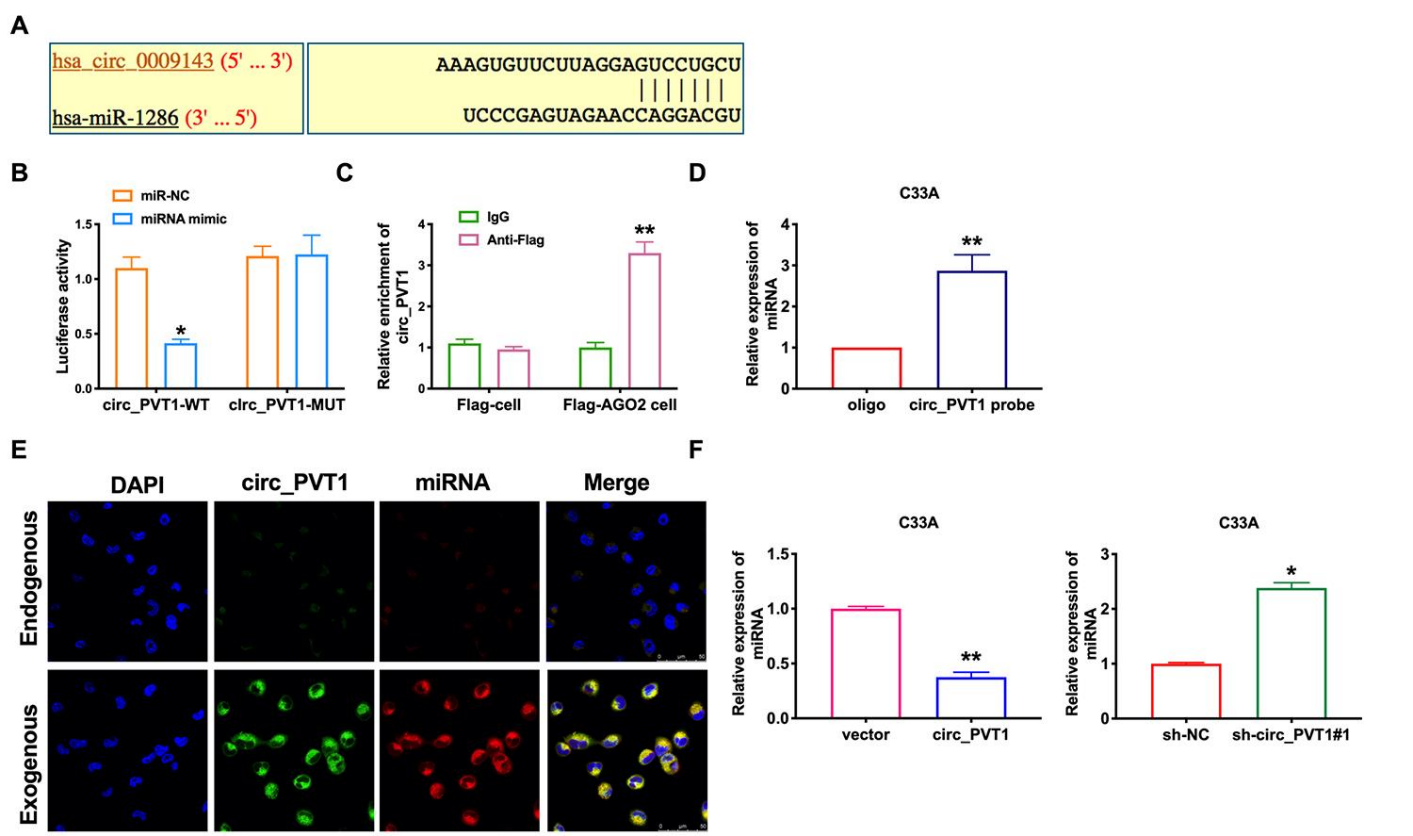
**Circ_PVT1 binds directly to miR-1286 in C33A cells.** (**A**) Schematic for predicting miR-1286 binding sites of circ_PVT1 (https://circinteractome.nia.nih.gov/api/v2/mirnasearch?circular_rna_query=hsa_circ_0009143&mirna_query=hsa-miR-1286&submit=miRNA+Target+Search). (**B**) The luciferase assay was performed to verify the interaction between miR-1286 and circ_PVT1. n=5, *P<0.05. (**C**) Binding situation between circ_PVT1 and miR-1286 verified using RNA pull-down. (**D**) The enrichment of circ_PVT1 was detected by RIP assay. n=5, **P<0.01. (**E**) Co-localization between circ_PVT1 and miR-1286 was revealed by fluorescence in situ hybridization. (**F**) The expression of miR-1286 were assessed in C33A cells. n=10, **P<0.01.

Because of its special molecular structure, circRNA has the functions of acting as miR-1286 "sponge", regulating transcription or splicing, and interacting with RNA binding proteins. It can also be used as a biomarker for disease diagnosis. Bioinformatics website predicts the existence of binding sites between circ_PVT1 and miR-1286 (Figure 4A). Next, we performed luciferase assays to confirm whether circ_PVT1 binds to miR-1286. The assay report showed that circ_PVT1 could bind with miR-1286 (Figure 4B). Further, we carried out anti-AGO2 RIP in C33A cells. Endogenous circ_PVT1 pull-down by AGO2 was enriched in cells transfected with miR-1286, revealing the direct binding of circ_PVT1 with miR-1286 (Figure 4C). Moreover, the results of RNA pull-down indicated an evident increase in the content of miR-1286 enriched by circ_PVT1 (Figure 4D). Therefore, the relationship of circ_PVT1 and miR-1286 was verified by FISH assay, and we found that miR-1286 was co-localized with circ_PVT1 in the cytoplasm, which once performed a relationship between circ_PVT1 and miR-1286 (Figure 4E). Then we detected the expression of miR-1286 in C33A cells after siRNA/plasmid transfection. After transfecting sh-circ_PVT1, the level of miR-1286 was up-regulated, and overexpression of circ_PVT1 inhibited the expression of miR-1286 (Figure 4F).

### MiR-1286 abolishes circ_PVT1-induced EMT and metastasis of cervical cancer cells

**Figure 5:**
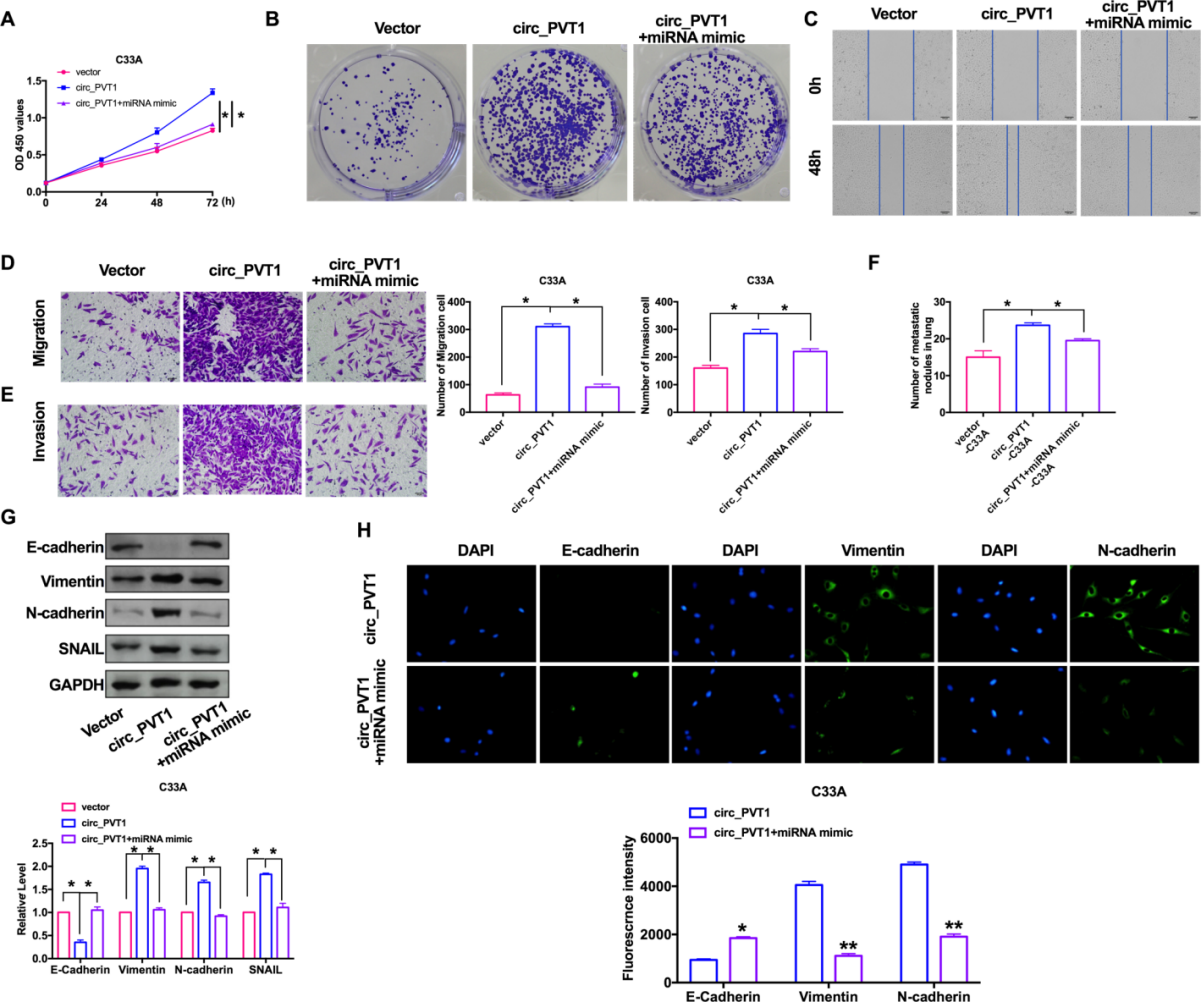
**MiR-1286 blocked circ_PVT1-induced EMT and invasion of cervical cancer cells.** (**A**) The CCK8 assay was performed in cervical cancer cell lines. n=8, *P < 0.05. (**B**) Clone formation assays in cervical cancer cell lines. (**C**) Wound healing assay was carried out for migration. (**D**, **E**) The migration and invasion were explored by transwell. n=6, *P<0.05. (**F**) The number of nodules was statistics for detecting lung metastasis. n=6, *P<0.05. (**G**) The expression of metastasis-associated protein, E-cadherin, Vimentin, N-cadherin and SNAIL were detected. n=6, *P<0.05. (**H**) Immunofluorescence representative images of E-cadherin, Vimentin, N-cadherin. n=6, *P<0.05, **P<0.01.

Next, we would the role of miR-1286 in the regulation of tumor progression by circ_PVT1. CCK-8 and clone formation assay showed that overexpression of miR-1286 could inhibit the proliferation ability induced by circ_PVT1 (Figure 5A, 5B). The C33A cells were co-transfected with circ_PVT1/miR-1286 mimics, by performing wound healing and transwell assay. The effect of overexpression of circ_PVT1 on cell migration was blocked by miR-1286 mimics (Figure 5C, 5D). Similarly, miR-1286 abolished the circ_PVT1 induction effect of invasion (Figure 5E). Meanwhile, miR-1286 and circ_PVT1 co-stabling cells injection mice performed less nodules than circ_PVT1 cells injection (Figure 5F). The change of protein expression induced by Circ_PVT1 was also reversed by miR-1286 (Figure 5G). The immunofluorescence results showed similar results (Figure 5H).

### Downregulation of miR-1286 aggravated the program of cervical cancer cells

**Figure 6:**
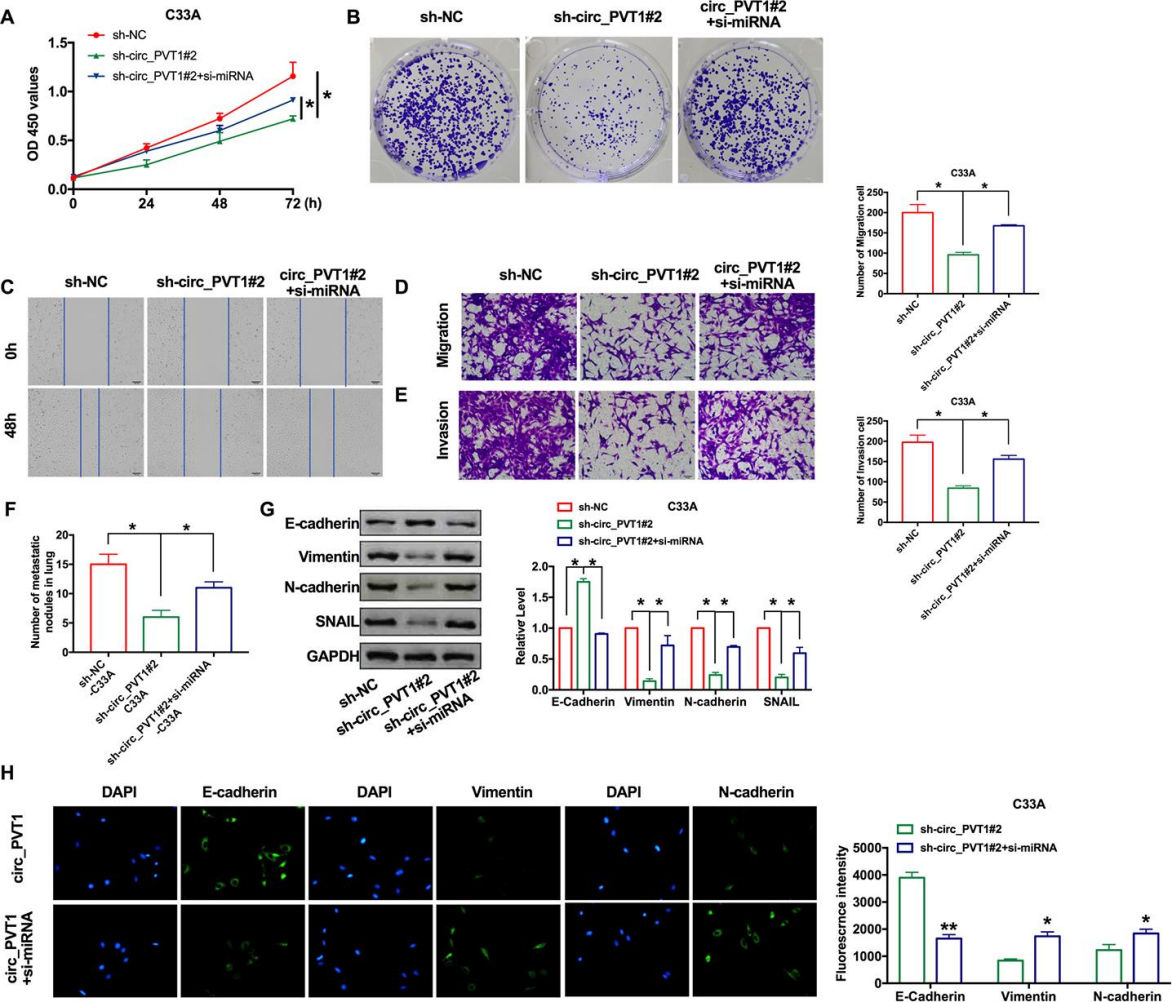
**Downregulation of miR-1286 induced EMT and invasion of cervical cancer cells.** (**A**) The CCK8 assay was performed in cervical cancer cell lines. n=8, *P < 0.05. (**B**) Clone formation assays in cervical cancer cell lines. (**C**) Wound healing assay was carried out for migration. (**D**, **E**) The migration and invasion were explored by transwell. n=6, *P<0.05. (**F**) The number of nodules was statistics for detecting lung metastasis. n=6, *P<0.05. (**G**) The expression of metastasis-associated protein, E-cadherin, Vimentin, N-cadherin and SNAIL were detected. n=6, *P<0.05. (**H**) Immunofluorescence representative images of E-cadherin, Vimentin, N-cadherin. n=6, *P<0.05, **P<0.01.

Our previous findings revealed that circ_PVT1 knockdown inhibits EMT and CC cells invasion. Considering the present findings that miR-1286 repressed circ_PVT1-induced EMT and invasion of cervical cancer cell**s**, we then hypothesized that miR-1286 may perform the similar phenotype caused by circ_PVT1 in C33A cells. The cells were co-transfected with siRNA/sh-circ_PVT1, as Figure. 6A and 6B shown, knockdown of miR-1286 could recovery the proliferation ability after sh-circ_PVT1 transfection. The inhibitory effect of sh-circ_PVT1 on cell migration was blocked by si-miR-1286 (Figure 6C, 6D). The suppression invasion of sh-circ_PVT1 was inhibited by miR-1286 downregulation (Figure 6E). *In vivo,* lung metastasis assay revealed si-miR-1286 and sh-circ_PVT1 co-stabling cells injection mice had more nodules than sh-circ_PVT1 cells injection (Figure 6F). The immunofluorescence staining of E-cadherin, Vimentin and N-cadherin all performed the inhibition function of si-miR-1286 on sh-circ_PVT1-surpression EMT and invasion (Figure 6G, 6H). Taken together, circ_PVT1 targeting miR-1286 regulated migration and metastasis of cervical cancer via controlling EMT program.

### Exosomal circ_PVT1 activates EMT *in vitro*

**Figure 7:**
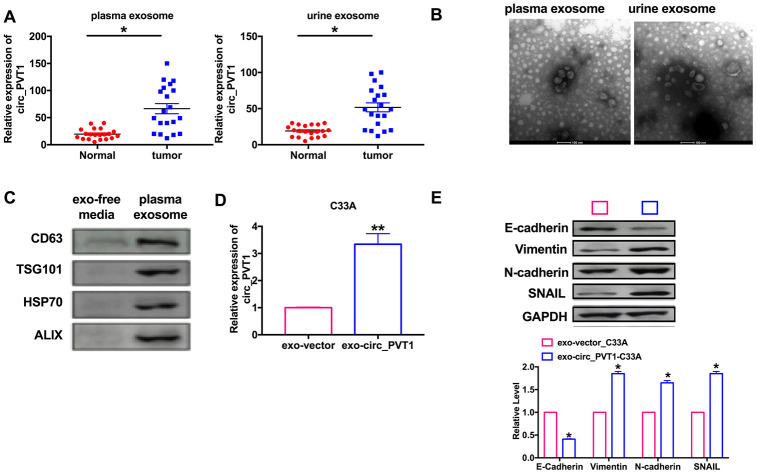
**Circ_PVT1 regulated EMT in CC cells via exosome pathway.** (**A**) The expression of circ_PVT1 was detected in exosome. n=20, *P<0.05. (**B**) TEM was used to determine the existence and morphology of exosomes from plasma and urine. (**C**) The biomarker (CD63, TSG101, HSP70 and ALIX) of exosome were detected in exosome-free media and exosome from plasma. (**D**) The expression of circ_PVT1 was detected in exosome from circ_PVT1/vector transfection cells. n=5, **P<0.01. (**E**) The expression of metastasis-associated protein, E-cadherin, Vimentin, N-cadherin and SNAIL were detected in exo-circ_PVT1/exo-vector co-cultured with C33A cells. n=5, *P<0.05.

Many circRNAs affect tumorigenesis and development through exosome pathway. We explored whether circ_PVT1 regulated CC migration and invasion via exosome pathway. we isolated exosomes in plasma and urine from 20 CC patients and 20 healthy volunteers, then we found the significantly higher level of circ_PVT1 in CC patients plasma and urine (Figure 7A). Then, we used a transmission electron microscope (TEM) to observe morphology of exosomes isolated plasma and urine (Figure 7B). Next, we detected the exosome biomarker CD63, TSG101, HSP70 and ALIX in exosome-free media and exosomes isolated from plasma, western blot results verified the exosome (Figure 7C). Then we transfection circ_PVT1 and vector into C33A cell and isolated exosome, then we detected that the exosomes from overexpression circ_PVT1 (exo-circ_PVT1) contained higher circ_PVT1 than exosome from vector transfection cells (exo-vector) (Figure 7D). The results indicated that circ_PVT1 could enter into exosome from C33A cell. Next, we co-cultured exo-circ_PVT1/exo-vector with C33A, we detected EMT biomarker E-cadherin, Vimentin, N-cadherin and SNAIL in co-cultured system. The down-regulated of E-cadherin and up-regulated of Vimentin, N-cadherin, SNAIL in exo-circ_PVT1-C33A system revealed that circ_PVT1 could induce EMT by exosome pathway (Figure 7E).

## DISCUSSION

In this study, we found that circ_PVT1 was upregulation in CC patients. Overexpression of circ_PVT1 promoted migration and invasion via EMT pathway. Knockdown of circ_PVT1 prevented migration and invasion via EMT pathway. Then we uncovered a new mechanism that circ_PVT1 sponges miR-1286 to affect EMT and eventually acts as a tumor promotor in CC via exosome pathway.

Cervical cancer is the most common cancer in the female reproductive system in China. In recent years, with the promotion of cervical cancer screening, the incidence of cervical cancer has been significantly reduced. With the improvement of the understanding of cervical cancer, cervical cancer is expected to be eliminated in the future. Targeted therapy has always been a hot research field, which has brought more new ideas and exploration to the treatment of cervical cancer.

The EMT process is very complex, which can be induced by multiple extracellular signals, which will gather in different gene families to activate a variety of EMT transcription factors [25]. With the deepening of the study of non-coding RNA, circRNA is considered to be an efficient and specific regulator of various EMT, which in turn controls the expression and function of circRNA through mutual feedback loops. Yang Z et.al GRSF1-mediated MIR-G-1 promotes EMT by directly increasing TMED5 and LMNB1 in cervical cancer cells [26]. iASPP/miR-20a could induce EMT and cisplatin resistance via FBXL5/BTG3 signaling pathway [27]. Circ_HIPK3 induced EMT of cervical cancer via targeting mir-338-3p [28]. CircPTK2 regulated growth and metastasis of CRC through controlling EMT pathway [29]. In this study, we found that circ_PVT1 induction of EMT to promote metastasis of cervical cancer cells

Tumor exosome is the pathfinder of tumor metastasis and plays a variety of roles in tumor metastasis. it includes determining the direction of tumor metastasis, transmitting tumor metastasis signals, promoting tumor cell migration, inducing epithelial-mesenchymal transformation, establishing pre-metastasis microenvironment and infectious metastasis behavior. Exosome will become a new target for the prevention and treatment of tumor metastasis. Circ-IARS promoted tumor metastasis via exosome secretion [30]. Exosomal-PDE8A induces cell development via the miR-338/MACC1/MET pathway in pancreatic cancer [31]. In our research, we found circ_PVT1 could enter into exosome from CC cells, and function as a tumor-promoter. There is no doubt that circ_PVT1 containing exosomes play an important role in cervical cancer metastasis and provide a new target for the prevention and treatment of tumor metastasis.

## MATERIALS AND METHODS

### Clinical samples

The tumor samples were collected from cervical cancer patients at Jinan City People’s Hospital. All of the patients or their guardians provided written consent. This study was approved by the Medical Ethics Committee of the Jinan City People’s Hospital and met the standard set in the Declaration of Helsinki.

### Cell culture

The cell lines (HUCEC, C33A, HCC-94, Hela and CASKI, C33A, HCC-94, Hela and CASKI were human cervical cancer cell lines. HUCEC was human normal cervical epithelial cells,) were purchased from the Science Cell Laboratory. Cell lines were cultured in PRIM 1640 (Thermo-life, United States) with 10 % FBS (Thermo Fisher, USA) and 100 μL/mL penicillin and streptomycin (Beyotime, China) and placed at 37°C with 5% CO2.

### Cell transfection

sh-RNA was produced by Ribobio Co., Ltd. (Guangdong, China). sh-circ_NC (negative control) was indicated as control (sh-NC). About 5×105 cells per well were seeded in 6 well plates, Regents (20 nmol/L) were transfected into the cells with lipo 2000 for 48 h.

### FISH

The subcellular localization of circ_PVT1 was detected using the FISH kit. The cells slide was added with the circ_PVT1 probe hybridization solution labeled by Digoxigenin, while the antagonistic circ_PVT1 probe served as a negative control (NC). The slide was hybridized at 42 °C for 16 h and immersed in 2 × SSC, followed by subsequent immersion in 70% ethanol for 3 min and staining with 4′6-diamidino-2-phenylindole (DAPI) for 10 min. The slide was photographed under a confocal laser-scanning microscope to document the observations.

### RNA immunoprecipitation assay

RNA immunoprecipitation was carried out using anti-Ago2 or control anti-IgG Abs with Magna RIP RNA-Binding Protein Immunoprecipitation Kit (Millipore). Beads were washed and purified RNA was subjected to qRT-PCR analysis and determined circ_PVT1 and miR-1286 expression levels.

### Pull down assay

A total of 1 × 107 C33A cells were harvested, lysed and sonicated. The circ_PVT1 probe was used for incubation with C-1 magnetic beads (Life Technologies) at 25 °C for 2 h to generate probe-coated beads. Cell lysate with circ_PVT1 probe or oligo probe was incubated at 4 °C for one night. After washing with wash buffer, the RNA mix bound to the beads was eluted and extracted with a RNeasy Mini Kit (QIAGEN) for RT–PCR or real-time PCR.

### Western blot

Total protein was collected from cells with RIPA lysis Mix (Beyotime, China). Briefly, 60 μg protein extraction was loaded via SDS-PAGE and transferred onto nitrocellulose membranes (MILLIPORE, USA), then put them into 5% blocking solution for 2 h. The membranes were incubated with primary antibodies at 4 °C for one night. After incubation with secondary antibodies, the membranes were scanned using an Odyssey, and data were analyzed with Odyssey software (LI-COR, USA). E-cadherin (20874-1-AP,1:1000) Vimentin (10366-1-AP, 1:1000) and SNAIL (13099-1-AP, 1:1000) were purchased from Proteintech; N-Cadherin (#14215,1:500) was purchased from CST, GAPDH (60004-1-Ig, 1:2000) was used as an loading control.

### Real time-PCR

Total RNA was isolated from cells according to a standard protocol. And then, the purity and concentration of RNA was detected and all the samples were converted into cDNA using reverse transcription kit. We used SYBR Green (Thermo Fisher Scientific) system to perform the qRT-PCR. Data was analyzed by GraphPad 7.

### Wound healing assay

Cells were seeded into 6-well plates at a density of 2.5 × 104 cells/cm2, and the 10 μL scratch was prepared. Subsequently, the samples were washed with phosphate-buffered saline (PBS) two times and incubated in the PRIM 1640 containing 10% FBS in a 5% CO2 incubator with saturated humidity at 37 °C. Images were obtained at 0 h and 48 h under an inverted microscope and analyzed using Image J software. The distance between the cells on both sides of the scratch at each time point (μm) was recorded. The migration distance of cells was calculated as an equivalent obtained by subtracting the distance between the scratch edge at 0 h from the migration edge at 48 h, which indicated the migratory ability of cells.

### Matrigel invasion assay

Cells in logarithmic growth phase were adjusted to 2 × 105 cells/well of medium (without serum) and plated 1μg/μl Matrigel into upper chamber. Lower chamber was added with 500 μL of medium, and then incubate the plate at 37 °C for 48 h. Then the invading cells were visualized by the crystal violet and inverted microscope.

### Luciferase assay

C33S cells were co-transfected with 20 mmol/L miR-1286 mimic or miR-NC together with circRNA-WT or circRNA-mutation. Luciferase activity was measured with Dual Luciferase Reporter Assay Kit (Transgene, China) on GloMax20/20 at 48 hr after the transfection.

### Immunofluorescence staining

Cells were plated in a 24-well cell culture plate. Cells were washed by PBS and fixed with 4% paraformaldehyde. Cells were permeabilized with 0.2% Triton-X-100 solution in PBS. Next, we blocked cell using goat serum. Then, the cells were incubated with E-cadherin (20874-1-AP,1:200) Vimentin (10366-1-AP, 1:100) and N-Cadherin (#14215,1:50) antibody at 4 °C overnight followed with FITC-conjugated goat anti-mouse antibodies incubation for 1h. After three washes with PBS, we incubated cells by DAPI.

### Lung metastasis assays

SPF grade BALB/c- nu/nu nude mice, 6 - 7 weeks and 20-24 g, were stably transfected with sh_PVT1_C33A/sh_NC_C33A cells. Each nude mouse was inoculated with 1 × 106 cells through the tail vein. According to the ethical method of experimental animals, the mice were killed and dissected, the transplanted tumor of the lung was removed, and the pulmonary nodules were calculated.

### Exosome isolation

The blood was collected in a 1.5mL tube and coagulated at 37 °C. Anticoagulant is not carried out from time to time. After that, the serum was obtained by centrifugation at 2000 × g for 10 minutes. (urine exocrine extraction is not carried out) then the serum was centrifuged with 3000 × g for 10 minutes. The supernatant was diluted with sterile PBS at 1:1 and centrifuged at 10000 × g for 30 minutes, followed by ultracentrifugation at 200000 × g for 2 hours. A large amount of PBS, washed in a is filtered through a 0.2-μ m syringe filter and centrifuged at 200000 × g for 1 hour, then the precipitates are collected and re-suspended in PBS or PBS medium for later functional or biochemical determination.

### Transmission electron microscope

The exosome was fixed. Washing with 1mL PBS for 3 times and rest 15 min each time, and fixing for 2 hours. Wash with 1mL PBS for 3 times and rest for 15 min each time. The 1mL gradient dehydration with 50, 70, 80, 90% ethanol was placed for 15 minutes respectively, and then dehydrated twice with 1mL 100% ethanol, each time 20min. 1mL was replaced with acetone for 2 times, and 15min was placed at rest each time. Put the sample into the embedding plate containing pure embedding agent. The embedded plate was polymerized at 65 °C for 48 h. Cleaning after 10min staining with uranyl acetate and cleaning after 10min staining with lead acetate. Then the samples were detected by transmission electron microscope.

### Statistical analysis

All values are expressed as the mean ± SEM. Statistical significances were measured by Student’s t-test and ANOVA A two-tailed value of P < 0.05 was indicated as statistically significant difference. Data statistics were used the GraphPad 7.0.
